# A cross-sectional study of the association between exposure to violence, intelligence, and executive function in Brazilian youths

**DOI:** 10.1186/s41155-023-00249-z

**Published:** 2023-02-27

**Authors:** Rhaná Carolina Santos, Nicole Prigol Dalfovo, Julia de Freitas Machado, Lucas Araújo de Azeredo, Rodrigo Grassi-Oliveira, Mirna Wetters Portuguez, Augusto Buchweitz

**Affiliations:** 1 BraIns — Brain Institute of Rio Grande do Sul, Av. Ipiranga, 6690, Porto Alegre, RS 90610-000 Brazil; 2 School of Life and Health Sciences, Pontifícia Universidade Católica do Rio Grande do Sul (PUCRS), Av. Ipiranga, 6681, Porto Alegre, RS 90619-900 Brazil; 3 School of Medicine, PUCRS, Av. Ipiranga, 6681, Porto Alegre, RS 90619-900 Brazil; 4 Translational Neuropsychiatry Unit, Department of Clinical Medicine, Aarhus University, Universitetsbyen 13, 8000 Aarhus C, Denmark; 5 Department of Psychological Science, University of Connecticut, Stamford, CT 06901 USA

**Keywords:** Working memory, Intelligence quotient, Victimization, Latin America, Adolescents

## Abstract

We carried out an exploratory study of the association between exposure to violence, intelligence, and executive functions in Brazilian preadolescents. The study included 56 participants (31 males) aged 8 to 14 years old (mean = 11.3, *SD* = 1.0). We administered neuropsychological tests to evaluate executive functions and the Juvenile Victimization Questionnaire (JVQ) to evaluate exposure to violence. We used the following neuropsychological instruments: Wechsler Abbreviated Scale of Intelligence (WASI), Stroop Color-Word Interference task, digits subtest of the Wechsler Intelligence Scale for Children, and an N-back task. We generated a composite score from neuropsychological test scores and investigated the association of that score, and individual test scores, with exposure to violence and socioeconomic status (SES). Results suggest, first, that exposure to violence is associated with a 0.5-point lower intelligence quotient score for every reported victimization event in the Juvenile Victimization Questionnaire. Results also show that the digits backward subtest scores showed a significant negative correlation with exposure to violence (JVQ; rho = −0.29, *p* < 0.05); both analyses were adjusted for the level of schooling of parents or guardians, which was also found to be significantly associated with lower intelligence quotient scores. We discuss results in the light of the existing literature on the effects of exposure to violence on adolescent development, and the amounting evidence that suggests an association of exposure to violence, and of victimization, with tests that evaluate constructs of executive functions. The study struggled with low compliance from participants, and we underscore the challenges of carrying out empirical studies aimed at better understanding the development of underrepresented youths, such as those from Central and Latin America.

**Supplementary Information:**

The online version contains supplementary material available at 10.1186/s41155-023-00249-z.

Violence impacts development and quality of life. There is robust evidence that early-life stressors affect cognitive and emotional development (Shonkoff et al., 2012; Pollak, 2015). The early-life stress that follows from trauma, maltreatment, and abuse on emotional and cognitive development is well-known owing to, e.g., studies of stress, early trauma, and institutionalization (Birn et al., 2017; Bremne and Vermetten, 2001; Rahdar and Galvan, 2014; Taylor, 2010). Stress and abuse are associated with outcomes that impact quality of adult life, such as greater likelihood of high-risk behaviors, unemployment, mood disorders, and drug use (Koppensteiner & Menezes, 2019; Stoddard et al., 2015). In terms of early life stress, a large cohort study of Brazilian children has linked childhood maltreatment to increased risk for disruptive behavior disorders (Bernardes et al., 2020), an outcome that significantly impacts quality of life. Likewise, the impact of chronic stress on adolescent development has been associated with lifelong neurodevelopmental and mental health outcomes, such as increased risk for mood disorders (Lupien et al., 2009; Eiland & Romeo, 2013).

Stress and its chronic forms may result from exposure to violence. Violence knowingly affects school performance, memory, and the ability to plan and carry out goals; these abilities are construed as attention and executive function (Dertelmann, 2011; Pechtel & Pizzagalli, 2011). The construct of executive functions allow for a bridge between the evaluation of underlying abilities and the prediction of their association with academic performance and quality of life. Violence also has negative effects associated with the development of socio-emotional skills, such as self-esteem and self-control (Tavares & Pietrobom, 2016). In sum, exposure to violence and being a victim of acts of violence impacts early and adolescent development to an extent that may affect early adulthood and quality of life.

Executive functions represent abilities that play a critical role in goal-oriented behaviors (Uehara et al., 2016; Seabra et al., 2014). It is postulated that the three main executive functions are inhibitory control, working memory, and cognitive flexibility (Diamond, 2013), which support our ability to plan, disregard distractions, hold and manipulate information critical to solving problems, and to make decisions. The development of executive functions are key to inhibiting distraction from one’s immediate goals and to direct attention to holding and manipulating information that helps decision-making. (Andrade & Flores-Mendoza, 2010; Mecca et al., 2014). Children are known for their shorter attention spans, as are adolescents; but with time, humans become better and better at their self-control and goal-directed behavior. Executive functions develop in childhood and adolescence, as do the brain networks that underpin this dynamic control of goal-directed behavior (Baum et al., 2020).

Adolescence is a formative, critical period for development of emotional and cognitive skills. It is also a period associated with an increased risk for exposure to violence and for physical and mental harm (Bustreo et al., 2013; Costello et al., 2003; Dahl & Suleiman, 2017; Patton et al., 2016). Relative to childhood, adolescence is associated with a remarkable increase in accidents, homicides, and substance use and an increase in risk for mental and eating disorders, suicide, sexually transmitted diseases, and unplanned pregnancy (Castellanos-Ryan et al., 2013; Kreipe & Birndorf, 2000; Paus et al., 2008). It is a time when the brain is flooded with desires while there it is still developing what little control there is (Shonkoff and Phillips, 2008). In this sense, stressors experienced in early adolescence, between 10 and 14 years of age, are strongly associated with a decrease in life expectancy, more so than stressors experienced, for example, during childhood (Dahl & Suleiman, 2017). Nonetheless, adolescence does represent a “second window” of opportunity for remediation and prevention of poor developmental and mental health outcomes (Dahl & Suleiman, 2017; Cará et al., 2019; Spear, 2013; Spielberg et al., 2014; Steinberg, 2008). But little is known (or, at least, relative to early stress, less is known) about the repercussions of exposure to violence in adolescents; to be sure, little is known about the impacts of stress and violence on youths in more vulnerable, low- and middle-income countries, such as Brazil (Pellizzoni et al., 2019; Willoughby et al., 2019). Yet, these youths are disproportionately affected by exposure to violence and stress (Cerqueira and Bueno, 2017). The goal of the present study was to investigate early adolescent exposure to violence and its association with measures of executive function and intelligence in Brazilian preadolescents, who are among the groups of youths disproportionately affected by violence but underrepresented in cognitive psychology studies of development.

## Method

The present study is part of an umbrella project, carried out between 2016 and 2019, which aimed to investigate exposure to violence as a mediating factor of differences in neural functions and structures as well as academic and cognitive performance in preadolescents. Participants were invited to participate in neuropsychological assessment, brain imaging, and hair cortisol concentration evaluations (Buchweitz et al., 2019; Cará et al., 2019). For the present study, we carried out a cross-sectional investigation whose data collection spanned across the years of 2018 and 2019.

### Participants

We invited participants who attended schools located in neighborhoods that had the highest rates of violence in an urban environment which, at the time of the study (years 2017–2018), was among the most violent in Brazil and in the world (Seguridad, Justicia y Paz. Consejo Ciudadano para la Seguridad Pública y la Justicia Penal, 2017). The study included 56 preadolescents (mean age 11.3 years; *SD* = 1.0; range 10–14 years; 31 boys). The inclusion criteria were the following: youths regularly enrolled in school, aged between 9 and 14 years, and literate (the questionnaire on exposure to violence requires individual, private reading of the questions); the exclusion criteria, in turn, were as follows: neurological or mental disorders and an intelligence quotient (IQ) below 70 (Wechsler Abbreviated Scale of Intelligence). Neurological or mental disorders were excluded based on the interview with parents or guardians. Table 1 presents descriptive demographic data for the sample. There were fewer records of the parent/guardian’s educational level (*n* = 54) and socioeconomic status (SES) (*n* = 52) than the total sample because demographic information was not obtainable for children who were wards of the state as a result of their parents having lost custody.

**Table 1:** Demographic data (*n* = 56)

Age (years), mean ± SD	11.3 ± 1.0
Sex, *n* (%)	
Male	31 (55.4%)
Female	25 (44.6%)
School year, mean ± SD	5.4 ± 1.1
Guardian schooling *n* (%)	
Illiterate/some elementary	9 (16.7%)
Elementary	13 (24.1%)
Middle	12 (22.2%)
Secondary	15 (27.8%)
Higher education	5 (9.3%)
SES *n* (%)	
B1/B2	14 (26.9%)
C1/C2	36 (69.2%)
D	2 (3.8%)

Parent/guardian schooling, (*n* = 54; 96.4%). Socioeconomic strata (*n* = 52; 92.9%) are based on a score derived from the Brazilian Association of Research Companies (ABEP) questionnaire. Using up-to-date (2020) strata, class D household earnings are, on average, between BRL $2090 and 4180 (roughly, US $380 to 760, as of June 2022 exchange rate); class C household earnings, from BRL $4180 to 10,450 for its C1 and C2 subdivisions (roughly, US $760 to 2000, as of June 2022 exchange rate); and class B, in their turn, BRL $10,450 to 20,900 (roughly, US $2000 to 4000, as of June 2022 exchange rate). According to the World Bank poverty line, D and most C families would be in extreme poverty. In a household of four, lower-income D class families earn approximately US $3.1 per head, per day; lower-income C families, about US $6.3 per head, per day; for reference, the World Bank poverty line for upper-middle-income countries is US $6.85, per head, per day (World Bank, 2022)

### Instruments and procedures

To explore the effects of exposure to violence on executive functions, we investigated constructs associated with executive functioning, including inhibitory control (Stroop Color-Word Interference task), attention and task switching (Trail Making Test), and working memory span (N-back and Digit span), and intellectual capacity, as construed by the intelligence quotient score (Wechsler Abbreviated Scale of Intelligence) (Heck et al., 2009). The tests were carried out in the schools, individually with each participant in a separate classroom made available by the school. Tests that require a psychologist for their application were carried out by psychologists and co-authors RCS, NPD, and JFM, and results were revised by MWP and AB. Tests were carried out in the following order: Stroop Color-Word Interference task (Stroop) (Scarpina & Tagini, 2017); Trail Making Test parts A and B (TMT A and B) (Bolfer, 2009); N-back auditory task (N-back) (De Nardi et al., 2013); Digit span from WISC-IV (Wechsler Intelligence Scale for Children) (Kaufman, 1994; Kaufman & Lichtenberger, 1999; Figueiredo & Nascimento, 2007); vocabulary subtest of WASI (Wechsler Abbreviated Scale of Intelligence) (Heck et al., 2009); and matrix reasoning subtest of WASI (Yales et al., 2006). Socioeconomic status (SES) was assessed using a standardized questionnaire (Associação Brasileira de Empresas de Pesquisa (ABEP), 2016), which was filled out by the parent or caregiver. The questionnaire provides a score based on household earnings, level of education and possession of consumer goods; approximately, 70% of the sample in the present study may be below the international poverty lines (see Table 1).

Exposure to violence was assessed using the reduced version of the Juvenile Victimization Questionnaire (JVQ) (Hamby et al., 2011), validated for Brazilian Portuguese by Da Silva (2017). It consists of 34 questions that can be presented to participants whose ages may range from 8 to 17 years. The JVQ collects information about types of victimization experienced; it provides a quantitative description of the main forms of crimes and infractions against youth: conventional crime, child abuse, peer and sibling victimization, sexual assault, and indirect witnessing and victimization (Finkelhor et al., 2005). The questionnaires were scored as one based on the item-level scores proposed by the manual. The module scores were used as dichotomous scores. Thus, a “yes” for a module indicated that at least one form of victimization on that module was reported, whereas a “no” indicated that no forms of victimization on that module were reported (see also Buchweitz et al., 2019; De Azeredo et al., 2020). The conventional crimes explore experiences of robbery, physical assault, being the victim of the use of force, kidnapping, and assaults due to gender, ethnicity, or religious belief; child abuse explores physical, psychological, and neglectful experiences against the child; peer and sibling victimization includes gang violence, bullying, or violent outcomes from dating; the sexual assault module involves sexual abuse, exposure to sexual content or scenes, and sexual harassment, practiced by strangers or known adults, young people of the same age, siblings, and friends; and finally, indirect witnessing and victimization investigates experiences of witnessing domestic violence, armed robbery, and shootings, for example. The present study was approved by the Ethics Committee of the Pontifical Catholic University of Rio Grande do Sul (PUCRS), project 7741516.6.0000.5336.

### Statistical analysis

The IQ was estimated using the Wechsler Abbreviated Scale of Intelligence (WASI), and scores were classified as very superior, superior, high average, average, low average, borderline, or extremely low according to the norms for this population (Trentini et al., 2014). We calculated a composite score for the executive function test scores including the following: Stroop Color-Word Interference tasks (in the third word list), Trail Making Test (score subtraction in part A from part B), Digit subtest (in backwards order), and auditory N-back task (third list). The scores from these tests were used to generate the score (or *z*-score). We calculated standardized (z) scores using the raw scores for Stroop Word-Color page, TMT parts B-A, N-back — third list, and Digit Backwards span. First, the means and standard deviations of each of these test sections were calculated for the sample of 56 subjects. From these data, *z*-scores were derived by the formula *z* = (x–x̅)/*SD*_sample_, where *x* is the raw score, *x̅* is the mean of the sample, and *SD* is the standard deviation of the sample. The standardized score was then obtained for each of the desired sections of the tests, for each of the subjects. The standardized scores were then averaged. Thus, we analyzed the association between exposure to violence (JVQ) and the composite *z*-score, for exposure to violence and each of four different executive function tests (i.e., Stroop Word-Color, TMT parts B-A, N-back — third list, and Digit Backwards span), and for exposure to violence and IQ. We also investigated correlations for the test scores and the composite score with demographic variables and for the multiple linear regression analyses.

Quantitative variables were described as mean and standard deviation or median and interquartile range, depending on the data distribution. Categorical variables were described by absolute and relative frequencies. We used Student’s *t*-test to compare means, and, in case of asymmetry, we used the Mann–Whitney *U*-test. We used Pearson’ or Spearman linear correlation tests were applied based on the normality of distribution of the data; we used the following notation for correlational analyses: *r* = Pearson correlation coefficient; rho = Spearman correlation coefficient. Finally, we used multiple linear regression model to investigate the relationship between the independent variable, JVQ score, and the dependent variables of test scores in executive function evaluations and IQ; we used guardian schooling as a covariable for the regression. We calculated the regression or angular coefficient (b), which measures the effect on the instruments of each 1-point increase, along with the 95% confidence interval. In addition, we calculated the coefficient of determination (*R*^2^) to assess the proportion of the variance in the neuropsychological evaluations that were explained by exposure to violence (JVQ-R2). We adopted the significance level of 5% (*p* < 0.05) and carried out the analyses using the *Statistical Package for the Social Sciences* (SPSS), version 29.0.

## Results

**Fig. 1:**
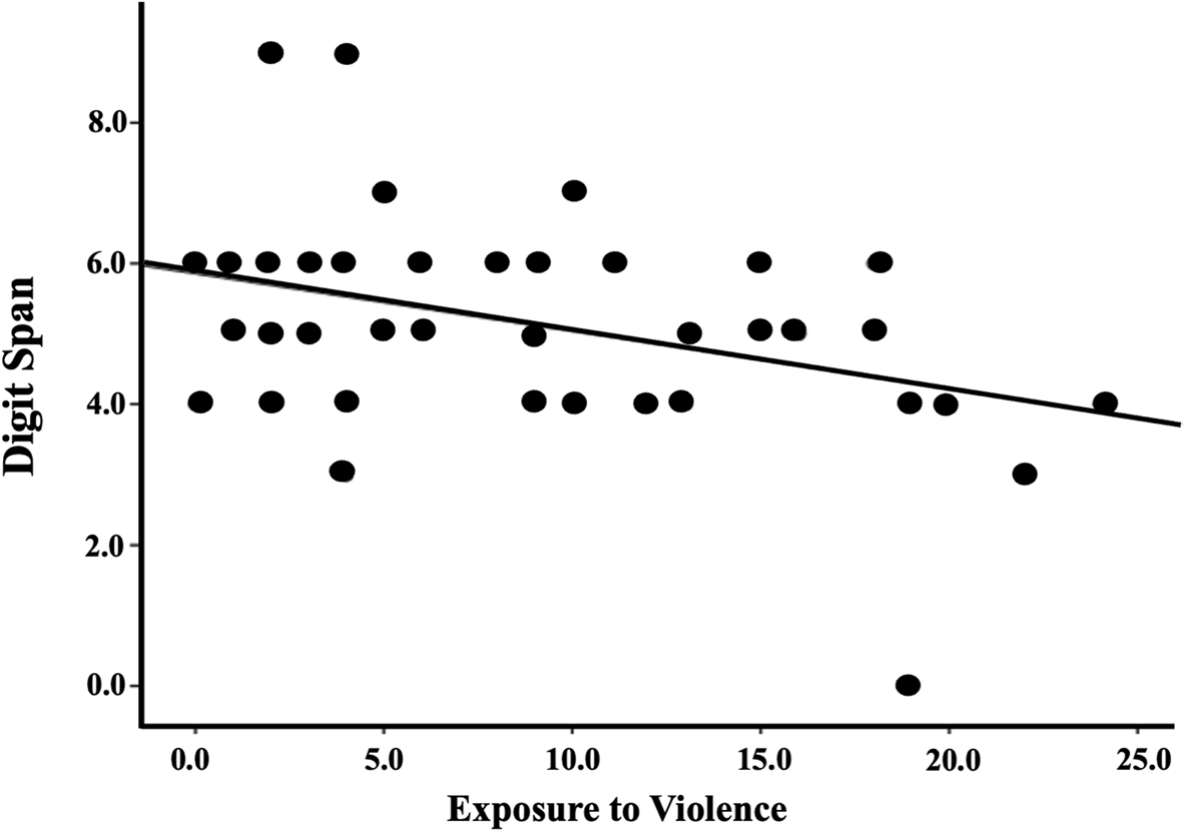
Correlation between digits backward and JVQ scores (note: correlation remains significant with removal of possible outlier — bottom of graph — with digits backward score near zero: *p* = 0.020)

The results showed a negative and statistically significant correlation between exposure to violence and the Digit Backwards span (rho = −0.29, *p* < 0.05; Fig. 1). The correlations between total exposure to violence and IQ, N-back, TMT, and the composite scores were not significant: IQ tests (rho = −0.259, *p* = 0.053), N-back sum (rho = −0.241, *p* = 0.073), TMT B-A (rho = 0.221, *p* = 0.101), and the composite score (rho = −0.226, *p* = 0.093). Multiple linear regression analysis showed a statistically significant association between exposure to violence and Digit Backwards span and IQ (Table 2). The greater the level or reported exposure to violence, the lower the Digit Backwards span and IQ scores. We chose to control for the effect of schooling of the parent or guardian since it showed a statistically significant correlation with the executive function scores.

**Table 2:** Multiple linear regression analysis for the association of JVQ score with executive function tests scores and JVQ score with IQ (adjusted by schooling of guardians)

	***b*** (95%CI)	***p***	***R***^**2**^
Digit span	−0.07 (−0.12 to −0.01)	0.022*	22.2%
IQ	−0.46 (−0.87 to −0.06)	0.026*	30.9%
Composite	−0.06 (−0.13 to 0.01)	0.069	12.4%
Stroop	0.20 (−0.23 to 0.64)	0.350	21.0%
TMT B-A	1.80 (−1.71 to 5.31)	0.309	18.7%
N-back	−0.35 (−0.73 to 0.03)	0.073	9.1%

Correlations showing *p* < 0.05 are highlighted in bold. Legend: *b*, slope (effect of JVQ on the variables); 95% *CI*, 95% confidence interval; *R*^2^, coefficient of determination (percentage of explanation of the model in relation to a specific outcome). Stroop, Stroop Word-Color; Digit span, Digit Backwards span; *TMT B-A*, Trail Making Test part B minus part A; N-back, sum 1A + 1B + 2A + 2B; Composite, composite (*z*-score). *The *p*-value corrected for multiple comparison for the regression with Digit Backwards span is *p* = 0.07, and for IQ, *p* = 0.07 (Benjamini & Hochberg, 1995)

Overall, 96.4% of the participants experienced at least one situation of victimization in their lives (lifetime prevalence). For a more in-depth discussion of the results on total exposure to violence, and the effects of experiencing two, three, or more violent events, “polyvictimized” youths, see de Azeredo et al. (2020) and Buchweitz et al. (2019). Only a quarter of parents or guardians had completed secondary education, and less than 1 in 10 had completed higher education.

There was an association between the parent’s or guardian’s schooling and social stratum and the youth’s Stroop test, Digits Backwards, IQ scores, and executive function composite scores. The descriptive statistics for executive function scores, IQ, and JVQ scores are shown in Supplementary Table 1(see, in turn, Supplementary Table 2 for the correlation between schooling, SES, and the test scores). There was no statistically significant correlation between the participant’s level of exposure to violence (JVQ) and the parent/guardian’s level of education; though only 2 participants were from low-income D SES level, according to Brazilian standards, most participants (*n* = 36) were from lower/middle-lower income, and a quarter were from middle-income families (see Table 1 for demographics and an explanation of the SES criteria for Brazil).

Finally, there were no statistically significant differences for exposure to violence (either total or to separate JVQ modules ) and sex (Supplementary Table 3). Further analyses of types of violence (JVQ modules) and neuropsychological tests suggest that maltreatment and sexual victimization were negative associated with scores in four of the tests, namely the Digit Backwards span, N-back, IQ, and executive function composite score tests (Supplementary Table 4). Moreover, the average IQ for the participants who reported sexual victimization was 86.6 (*SD* = 12.0), whereas the average for the participants who did not report sexual victimization was 94.8 (*SD* = 9.8). Thus, the average IQ for the subgroup of participants who reported sexual victimization was in the “low-average” range of the IQ norming for this demographic (low-average range 80.0 to 90.0); the average for the participants who were not a victim of sexual abuse is within the “average” range of IQ norms (average range 90 to 109) (Trentini et al., 2014). Participants who experience sexual victimization were also all polyvictimized, that is, they experienced three or more types of victimization. The mean JVQ scores for participants who reported sexual victimization were 15.6 (*SD* = 5.2); the mean for the entire group was 8.1 (*SD* = 6.3). Peer or sibling victimization, in turn, was negatively associated with the composite executive function score and IQ score. Witnessing violence did not individually show a statistically significant association with test scores (Supplementary Table 4).

## Discussion

The present study suggests that exposure to violence is significantly associated with lower scores in executive functions tests, i.e., digit span backwards, and in intelligence scores. Results show that for every experience of violence reported (i.e., one point in the JVQ score), youths scored half a point lower in the IQ test. The regression model suggests that exposure to violence explains 22.2% of the variability of Digit Backwards spans and 30.9% of the variability in IQ scores. Thus, for each point scored in the JVQ instrument, there was a 0.46-point reduction in IQ and a 0.07-point reduction in the Digit Backwards span. Maltreatment and sexual abuse, specifically, showed a significant negative correlation with the N-back and composite (*z*-score). This finding underscores that specific types of victimization may have a stronger impact on executive functions.

Research has shown a statistically significant association between mental health problems and the severity of multiple victimization (or polyvictimization) among Brazilian adolescents (De Azeredo et al., 2020). Understanding how exposure to violence and traumatic events can affect the developing executive functions may be highly informative for evidence-based policies that aim to change the course of developmental outcomes for adolescent populations, especially those living in the most violent environments but who are generally underrepresented in empirical studies of cognition and development.

Our findings corroborate studies that show impacts of trauma, violence, and stressful events on neuropsychological evaluation and tests (Cará et al., 2019; Augusti & Melinder, 2013; Fishbein et al., 2009). There were no significant differences for exposure to violence and sex of participants, but SES and parent/guardian’s level of schooling was correlated with test scores (but not with exposure to violence). Following more conservative analyses that included correction for multiple comparisons, the associations between exposure to violence and digit span backwards remained statistically significant. Hence, the strongest association to be had between violence and executive functions, in the present study, was between victimization scores and the ability to retain and manipulate information in the short term.

The significant negative association between violence and working memory is suggestive of impacts on a cognitive function that is critical for learning and attention, for example, which, in its turn, may impact educational and professional outcomes if the ability to maintain information and manipulate it is more permanently impaired (Sbicigo et al., 2013; Cará et al., 2019; Rahdar & Galvan, 2014, Bernardes et al., 2020). Relative to sex differences, in turn, though the number of male and female participants was similar (55.4% male), we did not identify a sex-related difference of exposure to violence in the present study. Nonetheless, we underscore that such differences have emerged in larger cohort studies, and that there is a growing body of evidence that shows gender disparities in, for example, the increased risk for intimate partner and for sexual violence among females (Stark et al., 2019; World Bank, 2015) and the higher risk for homicide among males (Fórum Brasileiro de Segurança Pública, 2020).

Adolescents can show behaviors and habits that include increased exposure to risks, increased risk-tasking behavior, and oppositionalism. These behaviors are expected and usually improve and can be remedied in early adulthood (Abranches & Assis, 2011; Dahl & Suleiman, 2017; Spear, 2013; Spielberg et al., 2014; Steinberg, 2008). Generally, the typical adolescent behavior need not impact development negatively. However, the combination of exposure to violence with such a period of “high power, low control” (Shonkoff & Phillips, 2008) can affect the emotional and cognitive development (Sbicigo et al., 2013; Cará et al., 2019; Dias & Seabra, 2013; Eiland & Romeo, 2013; Rahdar & Galvan, 2014). In this sense, the course of development of executive functions during this period can change as youths are faced with recurring experiences of violence (e.g., polyvictimized).

It is paramount to further understand the effects of violence on adolescence, the “second window” of development, for Latin American youths. The combination of education, SES, and environmental factors that put these youths at risk is increasingly alarming. Latin American are disproportionately affected by violence; they are more affected by violence compared to adults (Cerqueira & Bueno, 2017). The education and employment data for these youths also show some of the highest percentages of youths not in employment, education, or training (the ni-nis or NEET youths) (Novella et al, 2018). Moreover, the victims of violence and violent deaths in Brazil, as in other countries in the region, are getting increasingly younger. In 1980, the peak homicide rate affected victims who were 25 years of age; in 2017, the peak homicide rate dropped to an average 21 years of age (Cerqueira & Bueno, 2017). The homicide rate for Brazilian adolescents is four times the world average (and 93.9% of youth homicide victims are male) (Fórum Brasileiro de Segurança Pública, 2020)). Moreover, in Brazil, youths from lower socioeconomic strata are disproportionately affected by violence (Teixeira & Kassouf, 2015).

Of note, during 2020, there was an increase in violence — especially domestic violence — directly involving children and adolescents (Reinach & Burgos, 2021). The post-pandemic scenario has been wrought with more violence and poorer outlook for youths and young adults, e.g., with increasing numbers in school dropouts, unemployment, and NEET youths (not in employment, education, or training) (UNICEF, 2021; Cereda et al., 2020; Kiss et al., 2021).

## Conclusions

The present study was limited in its final sample size due to the loss of participants. We lost nearly two-thirds of the initial pool of participants (140 youths) due to voluntary withdrawal, inability to read and comprehend text, frequent absence from school, among other reasons (see also Buchweitz et al., 2019; Cará et al., 2019). Compliance in nonclinical studies of children and youth has been reported at approximately 78% (Wen et al., 2017). In this sense, future studies of lower- and middle-income countries may consider funding options that ensure participant adherence and, possibly, revise institutional review and ethics guidelines that prevent financial compensation to families. It is paramount to try to better accommodate underserved and lower-income families for participation in empirical studies; several families lack the time to accompany a minor and to participate.

## Supplementary Information

**Additional file 1: Supplementary Table 1.** Descriptive statistics for neuropsychological tests and IQ. **Supplementary Table 2.** Correlation between guardian schooling and SES with executive function tests and intelligence. **Supplementary Table 3.** Types of exposure to violence (median), total victims and victimization by sex (no significant differences between sexes). **Supplementary Table 4.** Correlation between types of exposure to violence and executive function tests and IQ scores.

## Data Availability

Supporting data are owned by the Brain Institute of Rio Grande do Sul. Restrictions apply to the availability of these data, which were used under license for the current study and are therefore not publicly available. However, the data can be made available by the authors upon reasonable request and with the permission of Dr. Augusto Buchweitz.

## Abbreviations

IQ: Intelligence quotient
JVQ: Juvenile Victimization Questionnaire
PUCRS: Pontifical Catholic University of Rio Grande do Sul
SES: Socioeconomic status
SPSS: Statistical Package for the Social Sciences
TMT: Trail Making Test
WASI: Wechsler Abbreviated Scale of Intelligence
WISC-IV: Wechsler Intelligence Scale for Children
