# Supplementary material for: A cross-sectional study of the association between exposure to violence, intelligence, and executive function in Brazilian youths
Source: Psicol Reflex Crit. 2023 Feb 27;36:6. doi: 10.1186/s41155-023-00249-z (PMC9968669; doi:10.1186/s41155-023-00249-z)
Supplement: Supplementary file 1 — Additional file 1: Supplementary Table 1. Descriptive statistics for neuropsychological tests and IQ. Supplementary Table 2. Correlation between guardian schooling and SES with executive function tests and intelligence. Supplementary Table 3. Types of exposure to violence (median), total victims and victimization by sex (no significant differences between sexes). Supplementary Table 4. Correlation between types of exposure to violence and executive function tests and IQ scores. [file 41155_2023_249_MOESM1_ESM.docx]

**Supplementary Table 1.** Descriptive statistics for neuropsychological tests and IQ.

| **Measure** | **Mean + SD [range]** |
| --- | --- |
| JVQ-Reduced* | 8.1 ± 4.3 [0 to 24] |
| Stroop | 26.8 ± 10.7 [9 to 54] |
| Digit span | 5.2 ± 1.4 [0 to 9] |
| TMT B-A | 111.7 ± 88.0 [-57 to 400] |
| N-Back | 15.5 ± 9.0 [0 to 38] |
| Composite | 0.30 ± 1.57 [-3.97 to 4.28] |
| IQ | 93.1 ± 11.0 [70 to 114] |

JVQ = Juvenile Victimization Questionnaire; SD = standard deviation; TMT B-A = Trail Making Test Part B minus Part A; IQ = Intelligence Quotient. Stroop = Stroop Word Color; Digit Span = Digit Backwards span; TMT B-A = Trail Making Test Part B minus Part A; N-Back = sum 1A+1B+2A+2B; Composite = Composite (z-score); * = Scores range from 0 to 34 points.

**Supplementary Table 2**. Correlation between guardian schooling and SES with executive function tests and intelligence.

|  | **Schooling, guardian** | | **SES** | |
| --- | --- | --- | --- | --- |
|  | rho (p)* | corrected p** | rho (p)* | corrected p** |
| IQ | **0.337 (0.013)** | **0.039** | **0.318 (0.022)** | **0.033** |
| Stroop | **0.313 (0.025)** | 0.050 | **0.354 (0.009)** | **0.018** |
| Digit span | **0.292 (0.038)** | 0.057 | **0.429 (0.002)** | **0.012** |
| TMT B-A | **-**0.087 (0.532) | 0.532 | -0.248 (0.076) | 0.091 |
| N-Back | 0.212 (0.124) | 0.148 | 0.097 (0.493) | 0.493 |
| Composite | **0.354 (0.009)** | **0.0.39** | **0.387 (0.005)** | **0.015** |

**Correlations showing p<0.05 are highlighted in bold**. Legend: Stroop = Stroop Word Color; Digit Span = Digit Backwards span; TMT B-A = Trail Making Test Part B minus Part A; N-Back = sum 1A+1B+2A+2B; Composite = Composite (z-score); SES = Socioeconomic stratum; * r = Pearson correlation coefficient; rho = Spearman correlation coefficient; **= p values corrected for multiple comparisons (Benjamini & Hochberg, 1995). There were no correlations for age of participants with test scores that resulted in p <0.05.

**Supplementary Table 3**. Types of exposure to violence (median), total victims and victimization by sex (no significant differences between sexes).

| **Victimization statistics by type of violence reported (JVQ module)** | | | | | | |
| --- | --- | --- | --- | --- | --- | --- |
|  | ***Lifetime prevalence of victimization*** | | | | | |
|  | **Median (P25–P75)** | **Victims** | | **Median by Sex** | |  |
|  |  | ***n*** | **%** | **M** | **F** | **p** |
| JVQ total score | 6 (3–13) | 54 | 96.4 | 8 (4–15) | 4 (2–11) | 0.257 |
| Conventional | 2.5 (1–4.8) | 48 | 85.7 | 3 (1–5) | 1 (1–4.5) | 0.304 |
| Maltreatment | 0 (0–1.8) | 24 | 42.9 | 0 (0–1) | 0 (0–2) | 0.352 |
| Peers or Siblings | 1 (0–3) | 39 | 69.6 | 1 (0–3) | 1 (0–2) | 0.317 |
| Sexual | 0 (0–1) | 16 | 28.6 | 0 (0–1) | 0 (0–1) | 0.975 |
| Witnessing | 2 (0.25–4) | 42 | 75.0 | 3 (1–4) | 1 (0–3.5) | 0.300 |

JVQ = Juvenile Victimization Questionnaire; n, number of victims according to the type of exposure to violence; % of the sample relative to n=56; Median by sex represents how much a type of exposure was reported by M, males and F, females; P25, 25^th^ percentile; P75, 75^th^ percentile. Conventional = Conventional Crimes; Peers or Siblings = Victimization by Peers or Siblings; Sexual = Sexual Victimization; Witnessing = Witnessing Violence and Indirect Victimization; p = P-value for the comparison across sex (Mann–Whitney *U* test).

**Supplementary Table 4**. Correlation between types of exposure to violence and executive function tests and IQ scores.

|  | **Type of Victimization Reported: rho (p)** | | | | |
| --- | --- | --- | --- | --- | --- |
|  | Conventional | Maltreatment | Peers/Sibs. | Sexual | Witnessing |
| Stroop | -0.010 | 0.065 | -0.024 | 0.06 | 0.13 |
| Digit span | -0.196 | **-0.382**** | -0.200 | **-0.438**** | -0.21 |
| TMT B-A | **0.307*** | 0.213 | 0.221 | 0.214 | 0.03 |
| N-Back | -0.206 | **-0.367**** | -0.182 | **-0.421**** | -0.11 |
| Composite | -0.156 | **-0.338*** | **-0.272*** | **-0.311*** | -0.14 |
| IQ | -0.172 | **-0.327*** | **-0.306*** | **-0.388**** | -0.13 |

Spearman correlation coefficients *p<0.05; **p<0.0. JVQ = Juvenile Victimization Questionnaire modules: Conventional = Conventional Crimes; Peers/Sibs. = Victimization by Peers or Siblings; Sexual = Sexual Victimization; Witnessing = Witnessing Violence and Indirect Victimization. Stroop = Stroop Word Color; Digit Span = Digit Backwards span; TMT B-A = Trail Making Test Part B minus Part A; N-Back = sum 1A+1B+2A+2B; Composite = Composite (z-score).
